# Causal association of calcific aortic valve stenosis and atrial fibrillation: a Mendelian randomization study

**DOI:** 10.1038/s41598-023-47770-w

**Published:** 2023-11-20

**Authors:** Chen Chai, Shoupeng Li, Lin Chen, Xiaobing Song

**Affiliations:** 1 Emergency Center, Hubei Clinical Research Center for Emergency and Resuscitation, Zhongnan Hospital of Wuhan University, No. 169, Donghu Road, Wuchang District, Wuhan, 430071 China; 2 Emergency Department, Wuhan Third Hospital (Tongren Hospital of Wuhan University), Wuhan, China; 3 Emergency Department, Xiantao First People’s Hospital Affiliated to Changjiang University, Xiantao, China

**Keywords:** Genetics, Cardiology

## Abstract

Calcific aortic valve stenosis (CAVS) is associated with an increased risk of atrial fibrillation (AF) in observational studies, but whether these associations are causal has not been determined. This study aimed to explore the potential causal relationship between CAVS and AF via Mendelian randomization (MR). Genetic variants from the genome-wide association study (GWAS) summary data of the European population for CAVS were used to investigate the association with AF. The inverse variance weighted (IVW) approach was used to obtain the primary causal inference, and several sensitivity analysis approaches, such as the MR‒Egger and weighted median (WM), were performed to assess the robustness of the results. A total of nineteen valid and independent genetic SNPs associated with CAVS were obtained from the GWAS database. Genetically predicted CAVS (OR: 1.105; 95% CI: 1.072–1.139; *p* = 8.60E−11) was associated with an increased risk of AF. Similar results were discovered in the sensitivity analyses by using MR Egger and weighted median approaches. An MR design was used to reduce confounding variables and the potential for reverse causality bias. The results provide genetic evidence that CAVS considerably increased the risk of AF.

Considering the worldwide aging trend and the development of wearable and smartphone-based devices for the monitoring of heart rate and rhythm, the prevalence of atrial fibrillation (AF) is growing globally^1^. According to the findings from the Framingham Heart Study (FHS), the prevalence of atrial fibrillation has tripled in the last 50 years^2^. The Global Burden of Disease project estimated that the number of patients with AF was very large, with approximately 46.3 million individuals in 2016^3^. This trend is predicted to continuously deteriorate in the future. For instance, the prevalence of AF in the European region in 2010 was about 9 million among individuals aged over 55 years old and is expected to reach 14 million by 2060^4,5^, and at least 72 million individuals in Asian areas will be diagnosed with AF by 2050^6^. Under these circumstances, targeted preventive measures need to be taken to prevent AF according to the risk factors for AF.

Several population-based studies have recently identified risk factors for AF, including left atrial (LA) enlargement, heart failure, valvular heart disease, hypertension, and aging^7–11^. Additionally, observational studies have revealed a potential relationship between AF and risk in patients at high risk for aortic stenosis (AS) ^12–16^. However, observational studies still have limitations due to confounders and reverse causality, making it challenging to assess the causal relationship between AS and AF.

Mendelian randomization (MR) is a method of using naturally occurring random allocation of genetic variation as a genetic instrument, with results similar to randomized controlled trials (RCTs) and less affected by confounding factors and reverse causality^17^. This study aimed to evaluate the causal relationship between CAVS and the risk of AF using MR methods.

## Methods

### Study design

MR analysis is based on three key assumptions: (1) exposure variables and genetic instrumental variation are consistently associated. (2) the outcome variables should not be associated with genetic instrumental variation. (3) only via the exposure variable can genetic instrumental variation significantly affect the outcome, but not in any other way.

**Figure 1:**
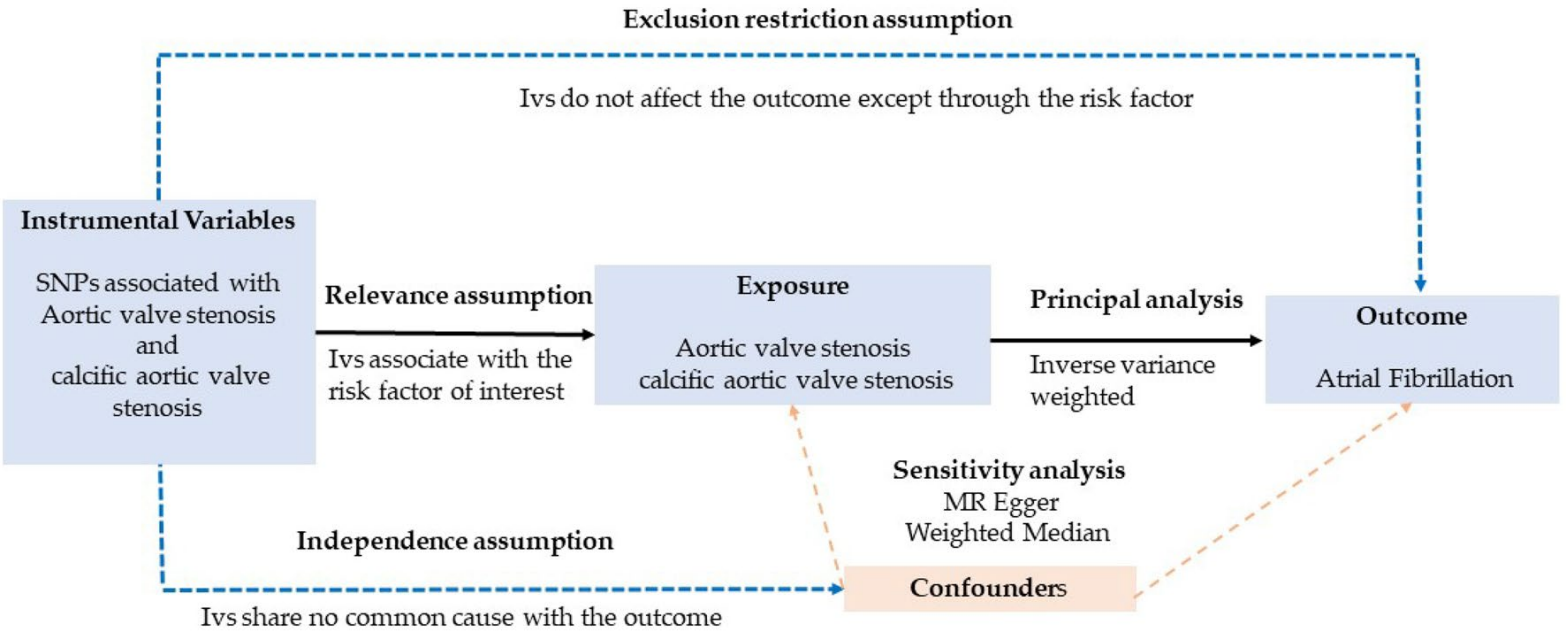
The overview of the study design.

An overview of the study design is shown in Fig. 1. We performed MR analysis using summary statistics from genome-wide association studies (GWAS). Genetic variants associated with CAVS were identified as instrumental variation, CAVS was used as the exposure and AF as the outcome, and the exposure and outcome datasets were harmonized to perform MR analyses, examining MR analysis assumptions and sensitivity analyses.

### Selection of instrumental SNPs

To select SNPs associated with CAVS as genetic instrumental variables for MR analysis, appropriate SNPs were selected based on a threshold of genome-wide significance (*p* < 5×10^−8^) and Linkage disequilibrium (r^2^ < 0.001). When harmonizing exposure and outcome data, palindromic SNPs that could lead to potential strand ambiguity were excluded. The F statistic was calculated to evaluate the strength of the instrument. F ≥ 10 indicates the association was not driven by weak instrument bias. This study included validated and independent genetic variants associated with CAVS as instrumental variables (see Supplementary Table S1 online).

### Data sources of calcific aortic valve stenosis and atrial fibrillation

The CAVS GWAS data from the FinnGen Project Database^18^, totaled 9153 cases and 368124 controls of European ancestry^19^. The FinnGen Project Database is a collaborative project led by the Institute for Molecular Medicine Finland (FIMM), the national biomedical infrastructure in Finland, which integrates clinical data, genetic data, and biological samples from all regions of Finland to create a large research resource.

Pooled data related to AF were obtained from a large GWAS meta-analysis study that included six contributing studies, namely, The Nord‐Trøndelag Health Study (HUNT), deCODE, Michigan Genomics Initiative (MGI), DiscovEHR, UK Biobank, and the Arial Fibrillation Genomics (AFGen) consortium^20^. A total of 60,620 cases and 970,216 controls of European ancestry. AF was diagnosed using ICD‐9 or ICD‐10 codes. Participants in the control group had diagnoses without any ICD-9 or ICD-10 codes. This study tested the relationship between AF and 34,740,186 genetic variations, identified 142 independent risk variants at 111 loci, and prioritized 151 functional candidate genes that potentially have an association with AF. The study design was described in detail in other publications^20^. The exposure and outcome information of GWAS summary data can be found in Supplementary Table S2 online.

### Statistical analysis

Two-sample Mendelian randomization (MR) was employed to assess the association between CAVS and AF. Three distinct two-sample MR approaches, including inverse variance weighted (IVW), weighted median (WM), and MR-egger, were utilized to evaluate the relationship between exposures (CAVS) and outcomes (AF). The IVW method, assuming no average pleiotropic effect, was considered the primary MR method for assessing potential causal effects^21^. Cochran’s Q test was initially performed to assess heterogeneity in individual causal effects, with *p* < 0.05 indicating the presence of heterogeneity. Subsequently, random-effect IVW was performed if heterogeneity was detected. Otherwise, the fixed-effect IVW method was adopted. The MR Egger approach was used to identify and assess directional pleiotropy effects in MR analyses, providing a sensitivity analysis for result robustness^22^. The WM approach required at least 50% of the weights to come from valid instrumental variables, enabling consistent estimates of causal effects and offering sensitivity analysis for the robustness of the results ^23^.

Sensitivity analyses were conducted to validate the causal estimates and account for potential confounding factors. Horizontal pleiotropy was assessed using the MR-Egger intercept term, where deviations from zero indicate the presence of directional pleiotropy. In the presence of horizontal pleiotropy, the slope of the MR-Egger regression provides unbiased MR estimations^22^. Furthermore, the stability of the causal estimates was evaluated through a leave-one-out analysis, where each SNP was sequentially excluded to assess the potential impact of a single SNP on the overall results.

Statistical analyses were conducted in R software (version 4.1.0, R Foundation for Statistical Computing) utilizing the TwoSampleMR (version 0.5.6) and Mendelian Randomization (version 0.5.1) packages. Multiple comparisons were performed using the Bonferroni correction (corrected *p* = 0.05/X/Y, where X denotes the number of exposures and Y denotes the number of outcomes). The observation of two-sided *p* < 0.025 was considered significant evidence for a causal effect, and *p* < 0.05 indicated significance.

## Results

### Calcific aortic valve stenosis and atrial fibrillation

Under the prior presumed standards, nineteen genetic variants associated with CAVS were obtained to explore the effect of CAVS genetic instrumental variables on AF. The characteristics of the genetic variants associated with CAVS and AF can be found in Supplementary Table S1 online. All of the F-statistics, which serve as an indicator of the instruments’ strength, exceed the conventional threshold of 10, indicating that the association was not driven by weak instrument bias.

As shown in Table 1, Cochran’s Q test for CAVS with AF revealed no evidence of heterogeneity (*p* > 0.05), and the fixed-effect IVW model demonstrated that genetically predicted CAVS (1.105; 95% CI: 1.072–1.139; *p* = 8.60E−11) is associated with an increased risk of AF. Similar results were discovered for both exposures in the sensitivity analyses by the MR Egger and WM approach. Moreover, the MR‒Egger regression intercept term showed no evidence of a directed pleiotropy effect between genetic variants (intercept, − 0.008; *p* = 0.142).

**Table 1:** The results of MR estimates of calcific aortic valve stenosis on atrial fibrillation, heterogeneity, and pleiotropy tests.

Exposure	Methods	OR	95% CI	*p*-Value	Cochran’s Q	df	*p* for Q	Egger intercept	*p* for pleiotropy
CAVS	IVW	1.105	1.072–1.139	8.60E−11	19.747	18	0.347	− 0.008	0.142
	MR Egger	1.169	1.082–1.262	9.95E−04	17.329	17	0.432		
	Weighted median	1.137	1.090–1.187	3.18E−09					

*OR* odds ratios; *CAVS* Calcific aortic valve stenosis; *IVW* inverse variance weighted.

**Figure 2:**
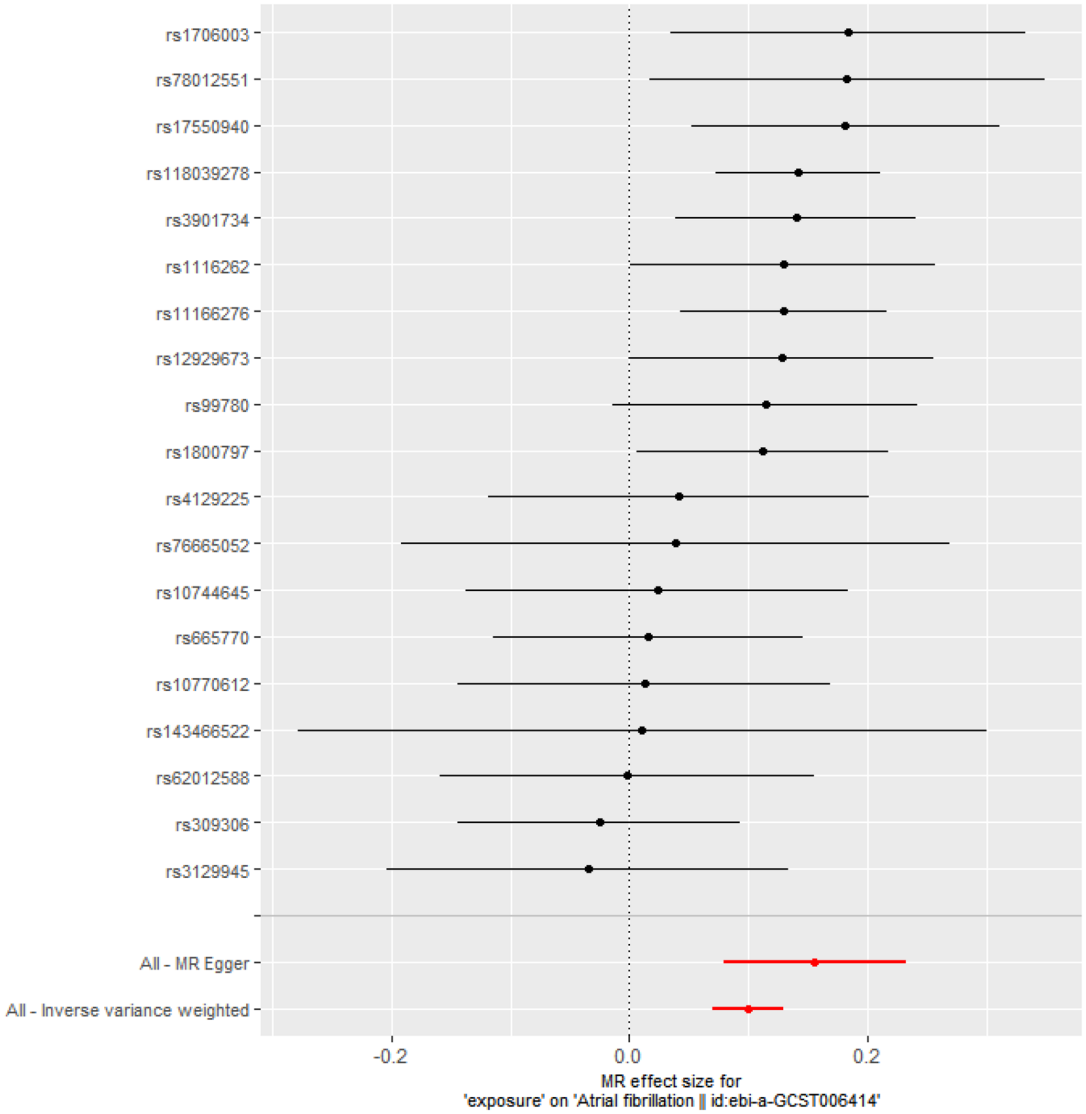
MR analysis for individual SNPs associated with calcific aortic valve stenosis in relation to atrial fibrillation risk.

Figure 2 shows the forest plots that represent the estimated single SNP effects of CAVS on AF. Eight SNPs (rs1706003, rs78012551, rs17550940, rs118039278, rs3901734, rs1116262, rs11166276, rs12929673, and rs1800797) among the selected nineteen SNPs in CAVS were significant for the estimated single effect on AF risk, whereas the other SNPs, e.g., rs143466522, rs4129225, rs76665052, etc., were not significant for the risk of AF.

**Figure 3:**
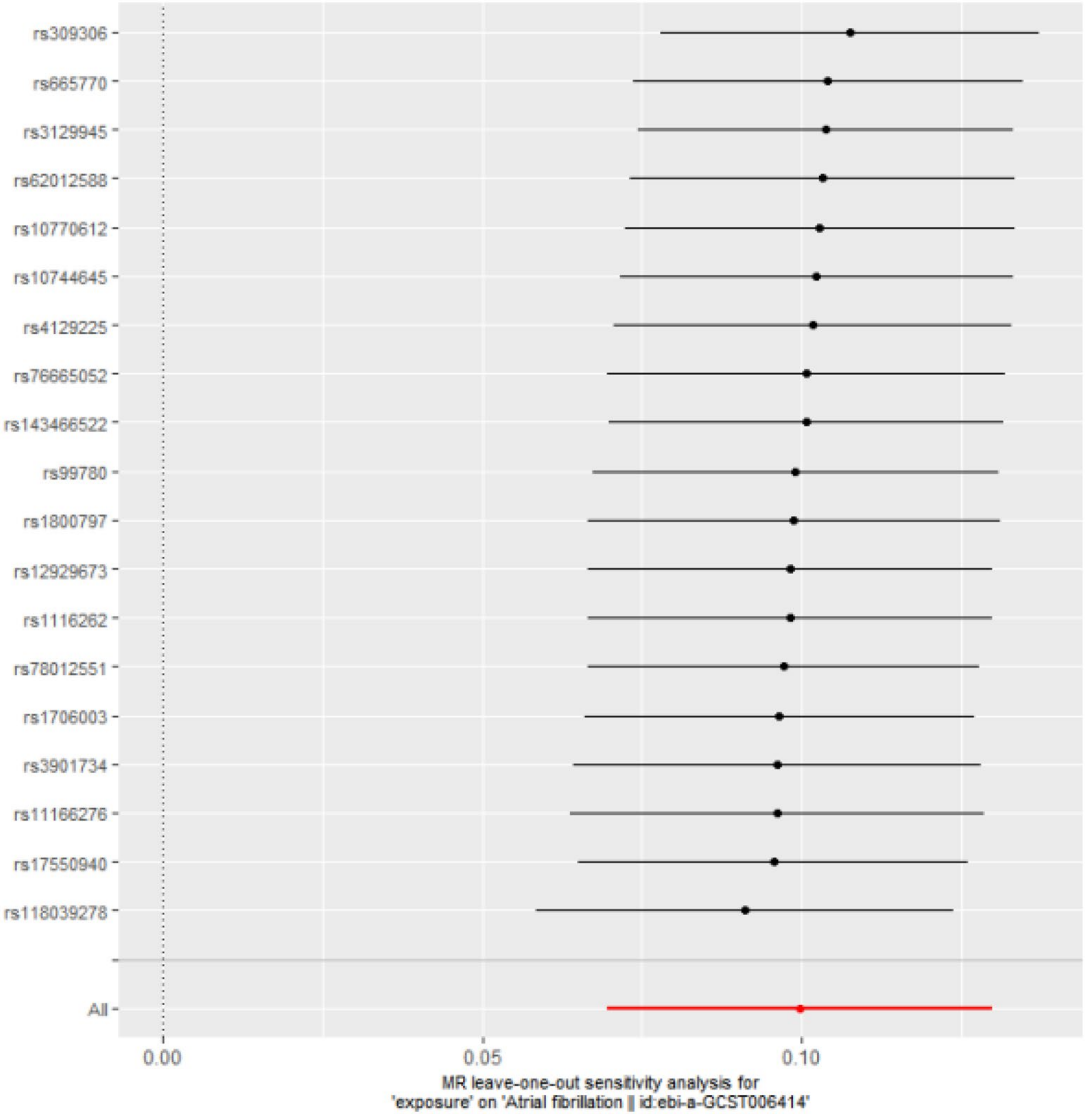
MR estimates between calcific aortic valve stenosis and atrial fibrillation by leaving one SNP out at a time.

The results of the leave-one-out analysis are illustrated in Fig. 3. In the leave-one-out analysis, no substantial change was observed in the genetically predicted risk estimates of CAVS with the risk of AF after excluding one SNP each time, indicating that the association of CAVS with AF was not driven by a single SNP, and the findings were stable.

### Sensitivity analysis

In addition, we conducted sensitivity analyses to assess the robustness of our findings by using two separate CAVS GWAS datasets. The first dataset included 13,765 cases and 640,102 controls of European ancestry^24^, while the second dataset involved 14,451 cases and 398,544 controls from the Million Veteran Program^25^(see Supplementary Table S2 online). The characterization of genetic variants associated with CAVS and AF in both groups is shown in Supplementary Table S1 online.

In the sensitivity analysis of CAVS-related SNPs and AF in both groups, we obtained consistent results with before (OR: 1.143; 95% CI: 1.107–1.181; *p* = 5.89E−16; OR: 1.194; 95% CI: 1.126–1.266; *p* = 3.49E-09), and Cochran's Q-test did not find heterogeneity evidence (*p* > 0. 05). Moreover, the MR-Egger regression intercept term showed no evidence of pleiotropic effect (intercept, − 0.908; *p* = 0.139; intercept,  − 0.0128; *p* = 0.213) (see Supplementary Table S3 online).

The forest plots of the single SNP effect estimates of CAVS on AF for the two groups are shown in Supplementary Fig. S1 online. The leave-one-out analysis of genetic variants of CAVS on AF for the two groups is shown in Supplementary Fig. S2 online.

## Discussion

Using two-sample Mendelian randomization, our data provided genetic evidence of a potential causal effect of CAVS on AF.

Our study shows that CAVS was associated with an increased risk of AF, which is consistent with previous observational studies. For instance, a population-based cohort study demonstrated a significant association between severe aortic valve stenosis and the risk of AF^26^. Similarly, a prospective study found that about one-third of patients without a history of AF who underwent Transcatheter Aortic Valve Implantation (TAVI) developed new-onset AF^12^. Furthermore, several other studies have reported that valvular heart disease increases the risk of AF by 1.8-fold and 3.4-fold in males and females, respectively^27^. The prevalence of AF was 9.1% in patients with mild to moderate AS and 33.7% in patients with severe stenosis^28,29^.

Mechanistically, there are several potential explanations. The accumulation of inflammatory cells in aortic valves is associated with remodeling and fibrosis ^24,30^, Alleles that increase the risk of AS are linked with elevated expression of IL6 and IL6-AS1 in fibroblasts^31^. On the one hand, fibroblasts, as the main cellular effectors of atrial fibrosis, contribute to AF by promoting fibrosis through the recruitment of inflammatory cells and excessive secretion of extracellular matrix (ECM) proteins^32^. Meanwhile, macrophages regulate fibrosis by producing proinflammatory cytokines (IL-6, ROS, and TNF-α) which can also lead to AF^33^. On the other hand, left atrial dilatation is believed to result from the stimulatory effect of IL-6 on matrix-metalloproteinase-2 (MMP2), a protease associated with atrial remodeling. Ultimately, IL-6 induces AF by inducing atrial remodeling ^34^. In addition, obesity, dyslipidemia, and calcification may contribute to aortic stenosis and be involved in the development of atrial fibrillation^11,30,35,36^. Pathophysiological studies have shown that AS can cause left ventricular outflow tract obstruction, resulting in left ventricular (LV) hypertrophy, and increases the left end-diastolic filling pressure resulting in left atrial (LA) dilatation, a key trigger of atrial fibrillation, which has been associated with the development of atrial fibrillation^37–41^.

This study provides genetic evidence that CAVS is associated with an increased risk of atrial fibrillation by using an MR design that minimizes confounding and reverse causation bias. We confirmed that the results were sufficiently robust by Sensitivity and multiplicity MR methods such as IVW, MR-Egger, and WM. Furthermore, there was no evidence of a directed pleiotropy effect between the genetic variants examined. Heterogeneity analysis revealed no significant variations among the SNPs studied. Additionally, leave-one-out analysis demonstrated that the overall effect was not driven by a single SNP, indicating the stability of our results.

Several limitations should be considered when interpreting our findings. Firstly, the use of summary-level statistics from published data prevented us from conducting nonlinear causal analyses. Secondly, the estimates of exposure and outcome were derived from aortic stenosis and atrial fibrillation cases obtained from linked hospital electronic health records, and the presence of comorbidities, disease progression, or severity was not evaluated. Lastly, the current study relies on genetic data collected from a predominantly European population, which, despite greater genetic homogeneity, limits the applicability of the current findings to other population groups.

## Conclusion

Our data provided genetic evidence supporting a possible causal relationship between CAVS and AF. Utilizing a two-sample MR design that minimizes confounding and reverse causality bias, we demonstrate an association between CAVS and an increased risk of AF.

### Supplementary Information

Supplementary Information.

## Data Availability

The data that support the findings of this study are available from the corresponding author upon reasonable request
