# Supplementary material for: Causal association of calcific aortic valve stenosis and atrial fibrillation: a Mendelian randomization study
Source: Sci Rep. 2023 Nov 20;13:20284. doi: 10.1038/s41598-023-47770-w (PMC10662195; doi:10.1038/s41598-023-47770-w)

Causal association of calcific aortic valve stenosis and atrial fibrillation: A Mendelian randomization study

Chen Chai, MS, [1#], Shoupeng Li, MS, [2#], Lin Chen, MS, [3#], and Xiaobing Song, Ph.D. [1*]

1. Emergency Center, Hubei Clinical Research Center for Emergency and Resuscitation, Zhongnan Hospital of Wuhan University, Wuhan, China

2. Emergency Department, Wuhan Third Hospital (Tongren Hospital of Wuhan University), Wuhan, China

3. Emergency Department, Xiantao First People's Hospital Affiliated to Changjiang University, Xiantao, China

Supplementary Materials

Supplementary Table S1. Characteristics of the genetic variants associated with calcific aortic valve stenosis and atrial fibrillation.

Supplementary Table S2. Information of GWAS summary data.

Supplementary Table S3. The results of MR estimate of calcific aortic valve stenosis on atrial fibrillation, heterogeneity, and pleiotropy tests.

Supplementary Figure S1. MR analysis for individual SNPs associated with calcific aortic valve stenosis about atrial fibrillation risk.

Supplementary Figure S2. MR estimates between calcific aortic valve stenosis and atrial fibrillation by leaving one SNP out at a time.

Supplementary Table S1. Characteristics of the genetic variants associated with calcific aortic valve stenosis and atrial fibrillation.

Exposures	SNP	Chr	Pos	EA	OA	EAF	R^2	*F*	Exposure			Outcome		
									beta	SE	*p*-value	beta	SE	*p*-value
CAVS-FinnGen Project Database	rs10744645	12	4495795	C	T	0.79	8.48E-05	32	0.11	0.02	1.53E-08	0.0026	0.009	0.774499
	rs10770612	12	20230639	G	A	0.24	8.23E-05	31	-0.10	0.02	2.50E-08	-0.0013	0.0083	0.8748
	rs1116262	6	160470880	A	G	0.01	0.000113	43	0.44	0.07	6.37E-11	0.0575	0.0291	0.04776
	rs11166276	1	100045239	T	C	0.48	0.000257	97	0.15	0.02	7.43E-23	0.0195	0.0067	0.003497
	rs118039278	6	160985526	A	G	0.05	0.000385	145	0.39	0.03	1.82E-33	0.0557	0.0139	6.22E-05
	rs12929673	16	75470295	T	C	0.56	0.00012	45	0.10	0.02	1.74E-11	0.0134	0.0068	0.04718
	rs143466522	19	11318472	A	G	0.01	8.78E-05	33	0.34	0.06	8.58E-09	0.0036	0.05	0.9425
	rs1706003	3	194299967	T	G	0.45	9.11E-05	34	-0.09	0.02	4.56E-09	-0.0167	0.0069	0.01576
	rs17550940	1	170657335	C	A	0.37	0.00013	49	0.11	0.02	2.58E-12	0.02	0.0073	0.006471

	rs1800797	7	22766221	G	A	0.47	0.000178	67	-0.13	0.02	2.67E-16	-0.0141	0.0068	0.03718
	rs309306	2	7160490	T	C	0.44	0.000134	50	-0.11	0.02	1.22E-12	0.0028	0.0067	0.6811
	rs3129945	6	32342537	A	G	0.25	9.16E-05	35	0.10	0.02	4.11E-09	-0.0036	0.0089	0.6857
	rs3901734	2	145769102	C	T	0.76	0.000171	64	0.15	0.02	1.01E-15	0.0207	0.0076	0.006715
	rs4129225	5	121948811	T	C	0.37	8.04E-05	30	-0.09	0.02	3.63E-08	-0.0037	0.0072	0.6083
	rs62012588	15	79032942	A	G	0.29	0.000111	42	0.11	0.02	1.01E-10	-0.0002	0.0086	0.9807
	rs665770	1	201748124	A	G	0.40	0.000115	43	0.10	0.02	4.45E-11	0.0016	0.0068	0.8171
	rs76665052	15	44968772	C	A	0.03	8.35E-05	32	-0.27	0.05	1.99E-08	-0.0107	0.0323	0.7406
	rs78012551	17	41711942	G	A	0.19	8.08E-05	31	0.11	0.02	3.33E-08	0.0194	0.009	0.0312
	rs99780	11	61596633	T	C	0.41	0.00012	45	-0.11	0.02	1.58E-11	-0.012	0.0069	0.082939
CAVS-European ancestry	rs10455872	6	161010118	G	A	0.07	0.000296	194	0.35	0.03	4.62E-44	0.0575	0.014	3.88E-05
	rs11643207	16	75498793	T	C	0.62	4.92E-05	32	0.08	0.01	1.41E-08	0.0116	0.0069	0.092521

rs117733303	6	160922870	G	A	0.02	9.75E-05	64	0.39	0.05	1.41E-15	0.0691	0.0256	0.007019
rs12740374	1	109817590	T	G	0.22	5.07E-05	33	-0.10	0.02	8.40E-09	-0.0154	0.008	0.05357
rs17156153	11	270514	T	C	0.08	5.28E-05	35	0.15	0.03	4.15E-09	0.0449	0.0121	0.000206
rs174533	11	61549025	A	G	0.35	5.14E-05	34	-0.10	0.02	6.66E-09	-0.0118	0.0069	0.089359
rs1800797	7	22766221	G	A	0.56	0.000116	76	-0.12	0.01	2.90E-18	-0.0141	0.0068	0.03718
rs551520	18	20094035	T	C	0.24	5.42E-05	35	-0.10	0.02	2.63E-09	-0.0025	0.0077	0.749799
rs55909255	9	106871788	C	T	0.39	4.57E-05	30	0.08	0.01	4.66E-08	0.0022	0.0068	0.746401
rs61817383	1	170665348	T	C	0.27	6.88E-05	45	0.10	0.02	1.99E-11	0.0241	0.0075	0.001205
rs62139062	2	65498898	T	G	0.27	4.59E-05	30	0.09	0.02	4.24E-08	0.0153	0.0075	0.04146
rs631556	1	201743185	A	G	0.40	6.3E-05	41	0.09	0.01	1.36E-10	0.0015	0.0068	0.8254
rs6696066	1	21872004	A	G	0.47	5.86E-05	38	-0.09	0.01	6.08E-10	-0.0124	0.0067	0.06312
rs6702619	1	100046246	G	T	0.51	0.000168	110	0.15	0.01	1.21E-25	0.0187	0.0067	0.005277

	rs6794263	3	153860779	C	A	0.11	5.02E-05	33	-0.13	0.02	1.01E-08	-0.0174	0.0104	0.092151
	rs7593336	2	145836429	A	G	0.61	9.26E-05	61	-0.11	0.01	7.26E-15	-0.0063	0.0068	0.3481
CAVS-Million Veteran Program	rs10455872	6	161010118	G	A	0.07	0.00055	227	0.35	0.02	2.6E-51	0.0575	0.014	3.88E-05
	rs11166276	1	100045239	T	C	0.49	0.000252	104	0.14	0.01	1.8E-24	0.0195	0.0067	0.003497
	rs117202424	6	118821706	A	G	0.05	0.000149	62	0.22	0.03	4.4E-15	0.0987	0.0158	4.51E-10
	rs12206973	6	118711303	C	G	0.05	0.000148	61	0.22	0.03	5.2E-15	0.1038	0.016	8.76E-11
	rs12740374	1	109817590	G	T	0.78	7.65E-05	32	0.10	0.02	1.9E-08	0.0154	0.008	0.05357
	rs1277930	1	109822143	A	G	0.77	8.18E-05	34	0.10	0.02	6.1E-09	0.0171	0.0079	0.03104
	rs1474347	7	22768124	C	A	0.42	0.000114	47	0.10	0.01	6.5E-12	0.0158	0.0067	0.01887
	rs1522387	3	57946096	A	G	0.57	7.5E-05	31	0.08	0.01	2.6E-08	0.0206	0.0067	0.002218
	rs1522388	3	57945768	T	C	0.56	7.5E-05	31	0.08	0.01	2.6E-08	0.0212	0.0067	0.001582
	rs174533	11	61549025	G	A	0.67	0.000153	63	0.11	0.01	1.7E-15	0.0118	0.0069	0.089359

rs2246363	2	145783282	A	G	0.75	0.000106	44	0.10	0.02	3.8E-11	0.0204	0.0077	0.007714
rs3753782	1	21879524	A	G	0.84	8.29E-05	34	0.11	0.02	4.9E-09	0.0206	0.009	0.02216
rs59030006	3	169207475	T	C	0.51	7.65E-05	32	0.08	0.01	1.9E-08	-0.0002	0.0067	0.9771
rs6493062	15	43017919	G	A	0.81	8.05E-05	33	0.10	0.02	8.1E-09	0.0059	0.0084	0.4804
rs742152	22	37896749	T	C	0.77	8.18E-05	34	0.10	0.02	6.2E-09	-0.006	0.0081	0.4612
rs7543130	1	100049785	A	C	0.5	0.000252	104	0.14	0.01	1.8E-24	0.0193	0.0067	0.003696

Abbreviations: CAVS, Calcific aortic valve stenosis; SNP, single nucleotide polymorphism; Chr, chromosome; Pos, position; EA, effect allele; OA, other allele; EAF, effect allele frequency; SE, standard error.

Supplementary Table S2. Information of GWAS summary data.

GWAS summary data	First Author	Sample size	Population ancestry	Reference
CAVS-FinnGen Project Database	NA	9153 cases and 368124 controls	European	https://r9.finngen.fi/pheno/I9_CAVS_OPERATED
CAVS-European ancestry	Yu Chen H et al.	13765 cases and 640102 controls	European	Yu Chen H., et al. Dyslipidemia, inflammation, calcification, and adiposity in aortic stenosis: a genome-wide study. Eur Heart J. 2023 Jun 1;44(21):1927-1939. doi: 10.1093/eurheartj/ehad142. PMID: 37038246; PMCID: PMC10232274.
CAVS--Million Veteran Program	Small AM et al.	14451 cases and 398544 controls	European	Small AM., et al. Multiancestry Genome-Wide Association Study of Aortic Stenosis Identifies Multiple Novel Loci in the Million Veteran Program. Circulation. 2023 Mar 21;147(12):942-955. doi: 10.1161/CIRCULATIONAHA.122.061451. Epub 2023 Feb 20. PMID: 36802703; PMCID： PMC10232274.
Atrial Fibrillation	Nielsen et al.	60620 cases and 970216 controls	European	Nielsen, J.B., et al., Biobank-driven genomic discovery yields new insight into atrial fibrillation biology. Nat Genet, 2018. 50(9): p. 1234-1239. doi: 10.1038/s41588-018-0171-3.PMID:30061737; PMCID: PMC6530775.

Abbreviations: CAVS, Calcific aortic valve stenosis.

Supplementary Table S3. The results of MR estimate of calcific aortic valve stenosis on atrial fibrillation, heterogeneity, and pleiotropy tests.

Exposure	Methods	OR	95%CI	*p*-value	Cochran's Q	df	p for Q	Egger intercept	p for pleiotropy
CAVS-European ancestry	IVW	1.143	1.107-1.181	5.89E-16	15.153	15	0.440		
	MR Egger	1.217	1.118-1.325	4.54E-04	12.69	14	0.551	-0.908	0.139
	Weighted median	1.144	1.093-1.198	9.42E-09					
CAVS--Million Veteran Program	IVW	1.194	1.126-1.266	3.49E-09	49.425	15	1.49E-05		
	MR Egger	1.311	1.127-1.525	3.51E-03	44.064	14	5.78E-05	-0.012	0.213
	Weighted median	1.176	1.119-1.236	2.10E-10					

Abbreviations: OR: odds ratios; CAVS, Calcific aortic valve stenosis; IVW: inverse variance weighted.

Supplementary Figure S1. MR analysis for individual SNPs associated with calcific aortic valve stenosis about atrial fibrillation risk.

CAVS-European ancestry

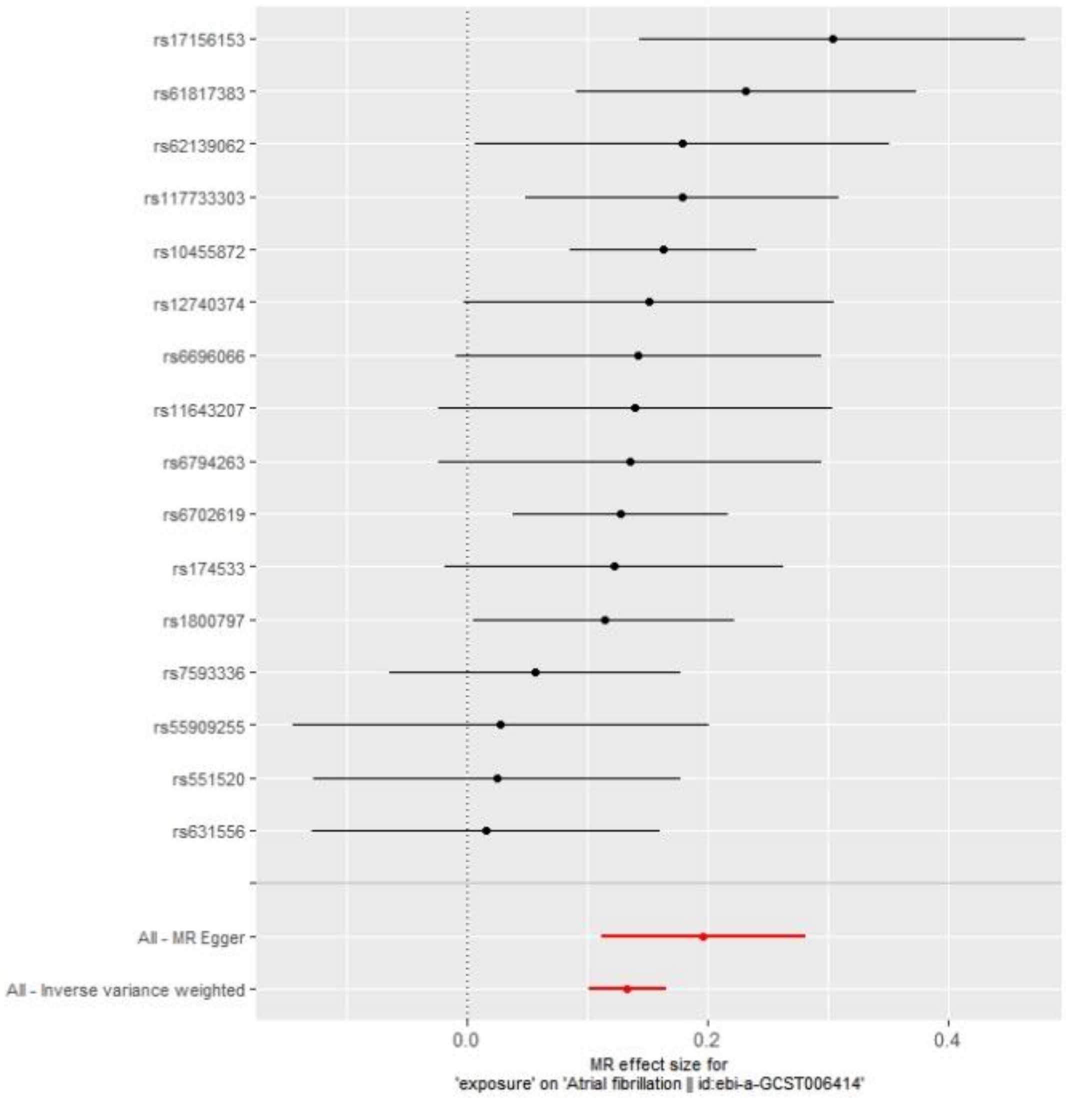

CAVS--Million Veteran Program

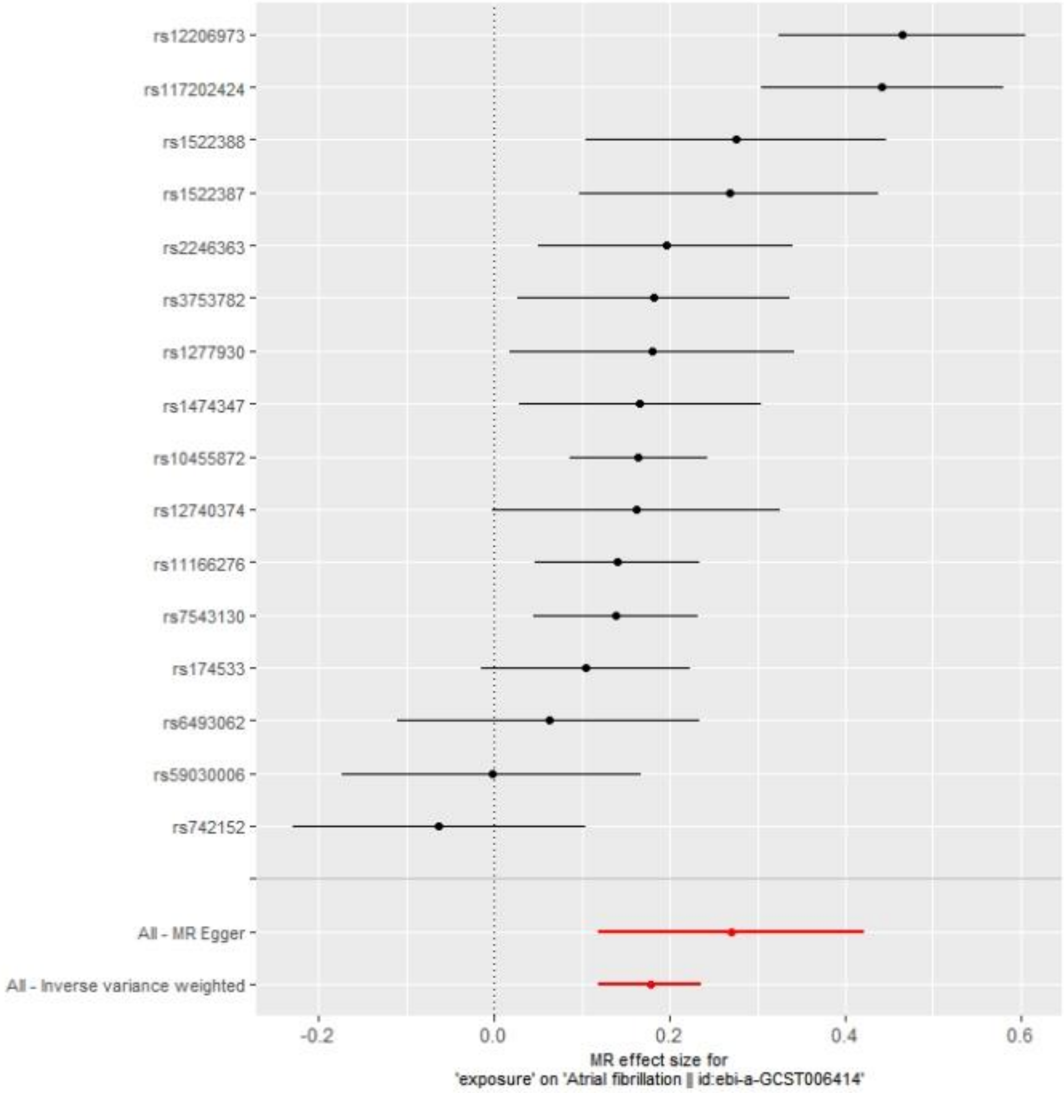

Supplementary Figure S2. MR estimates between calcific aortic valve stenosis and atrial fibrillation by leaving one SNP out at a time.

CAVS-European ancestry

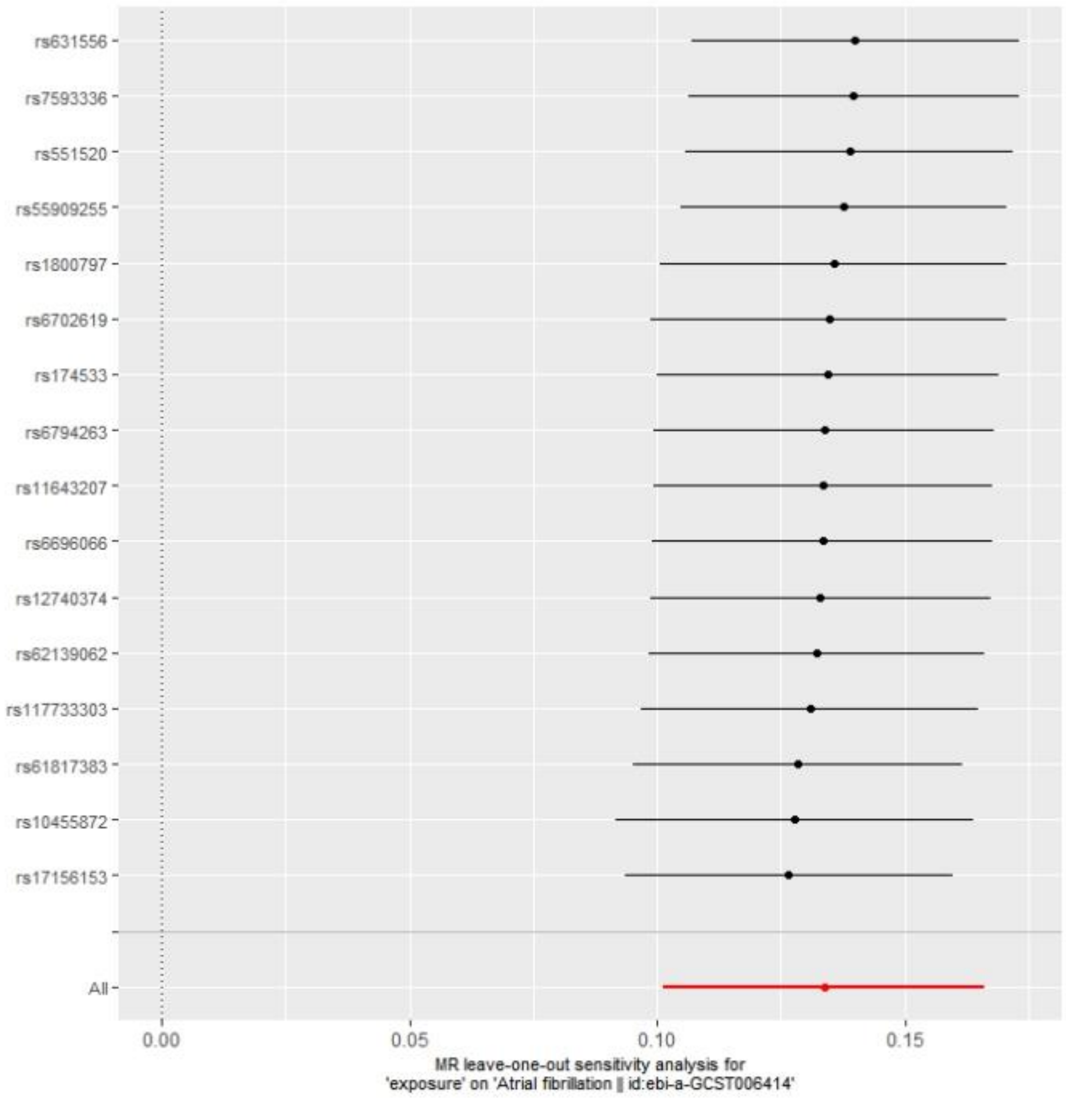

CAVS--Million Veteran Program

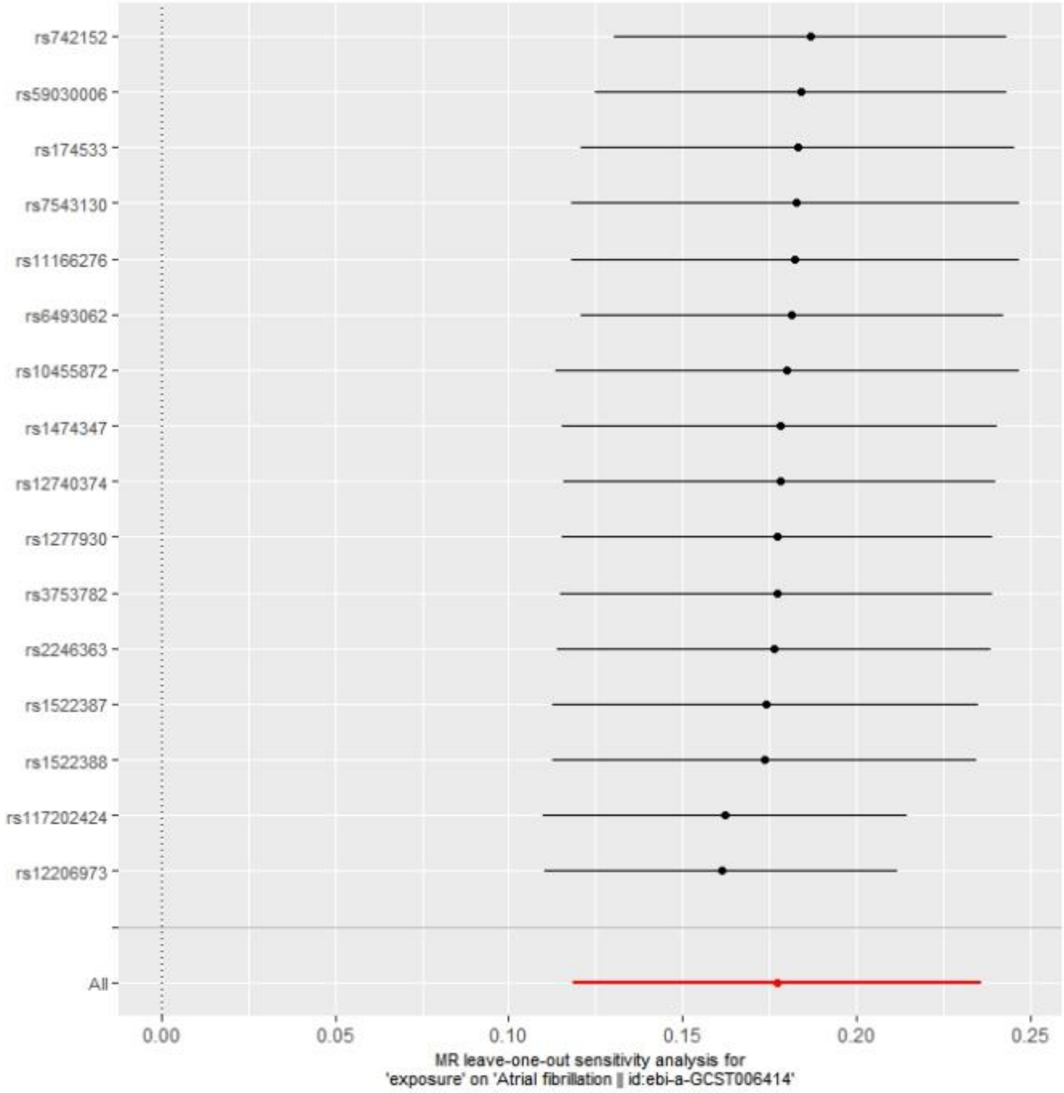

Supplement: Supplementary file 1 — Supplementary Information. [file 41598_2023_47770_MOESM1_ESM.pdf]
